# Anti-Diabetic Activities of *Gastrodia elata* Blume Water Extracts Are Mediated Mainly by Potentiating Glucose-Stimulated Insulin Secretion and Increasing β-Cell Mass in Non-Obese Type 2 Diabetic Animals

**DOI:** 10.3390/nu8030161

**Published:** 2016-03-11

**Authors:** Hye Jeong Yang, Min Jung Kim, Dae Young Kwon, Da Sol Kim, Young Hyun Lee, Ji Eun Kim, Sunmin Park

**Affiliations:** 1 Food Functional Research Division, Korean Food Research Institutes, Sungnam 463-746, Korea; yhj@kfri.re.kr (H.J.Y.); kmj@kfri.re.kr (M.J.K.); dykwon@kfri.re.kr (D.Y.K.); 2 Department of Food and Nutrition, Obesity/Diabetes Center, Hoseo University, Asan 336-795, Korea; tpfptm14@daum.net (D.S.K.); tjtnwls56@hanmail.net (J.E.K.); 3 Department of Nanobiomechatronics, Hoseo University, Asan 336-795, Korea; tppoc002@naver.com

**Keywords:** *Gastrodia elata* Blume, insulin secretagogues, β-cell mass, insulin resistance, type 2 diabetes

## Abstract

The brain is an important modulator of glucose metabolism, and is known to respond *Gastrodia elata* Blume water extract (GEB). Therefore, we examined whether long-term administration of GEB has hypoglycemic activity, and its action mechanism was explored in partially-pancreatectomized rats that exhibit similar characteristics as Asian type 2 diabetes, non-obese insulin-insufficient diabetes. The rats were provided high-fat diets supplemented with either of (1) 0.5% GEB (GEB-L), (2) 2% GEB (GEB-H), (3) 2% dextrin (control), or (4) 2% dextrin with rosiglitazone (20 mg/kg body weight; positive-control) for eight weeks. GEB dose-dependently improved hypothalamic insulin signaling, enhanced whole-body insulin sensitivity during hyperinsulinemic euglycemic clamp, and reduced hepatic glucose output in a hyperinsulinemic state. GEB dose-dependently increased the area under the curve of the serum insulin levels at the first and second phases during hyperglycemic clamp compared to the control, whereas the positive control had no effect. Insulin sensitivity during the hyperglycemic state also improved, dose-dependently, in response to GEB compared with that of the control, but was less than the positive control. GEB-H increased the mass of β-cells by potentiating proliferation and decreasing apoptosis. In conclusion, GEB could be a therapeutic agent for treating Asian type 2 diabetes.

## 1. Introduction

Type 2 diabetes is induced by an imbalance between insulin resistance and insulin secretion and does not occur unless β-cell function cannot compensate for insulin resistance [1]. Insulin resistance is the major pathophysiology for type 2 diabetes in Caucasians, as β-cell function is sufficient to overcome insulin resistance. However, patients eventually fail to compensate for insulin resistance because increased insulin secretion does not normalize serum glucose levels by reducing insulin resistance [1]. However, failure of β-cell function occurs more frequently in Asians than in Caucasians. Insulin secretion capacity is associated with β-cell mass, which tends to be lower in Asians [2]. Therefore, impaired β-cell function is a major risk factor for developing type 2 diabetes in East Asians. Therapeutic agents for Asian type 2 diabetes need to not only enhance insulin sensitivity but also potentiate β-cell function and mass.

*Gastrodia elata* Blume (GEB) is a plant in the Orchidaceae family that has been used traditionally to treat convulsions, ischemia, Alzheimer’s disease, tremors, and vertigo [3,4]. It contains many phenolic compounds, including gastrodin [4-(β-d-gucopyranosyl) benzyl alcohol], 4-hydroxybenzyl alcohol, 4-hydroxybenzaldehyde, 4-hydroxy-3-methoxy benzaldehyde, and vanillin, which are small phenolic compounds that can pass through the blood brain barrier (BBB) where they have antioxidant, anti-inflammatory, and anti-angiogenic activities to enhance neurological disorders, including ischemic stroke and Alzheimer’s disease [3,5,6]. Previous studies have demonstrated that Alzheimer’s disease is associated with brain insulin resistance, which is interrelated with type 2 diabetes [7,8]. Alzheimer’s disease is type 3 diabetes or brain-specific type 2 diabetes [7]. Moreover, the potentiation of insulin signaling in the hypothalamus and hippocampus improves peripheral glucose and energy metabolism. Thus, beneficial therapeutic agents against type 2 diabetes may be beneficial against Alzheimer’s disease, and *vice versa*. Therefore, GEB may be beneficial for preventing and alleviating the symptoms of type 2 diabetes.

However, GEB has not been studied for its therapeutic effect in type 2 diabetes. In our previous study, GEB decreased visceral fat mass by increasing fat oxidation and lowering food intake, resulting in enhanced insulin sensitivity in diet-induced obese male rats [9]. Vanillin and 4-hydroxybenzaldehyde increase insulin-stimulated glucose uptake by lowering fat accumulation in 3T3-L1 adipocytes [9]. However, the activity of these compounds to stimulate insulin-stimulated glucose uptake was much less than that of rosiglitazone in a cell-based study [9]. These results suggest that GEB may improve glucose metabolism by activating energy use in obese animals and that the improvement is related to indirect activation of the hypothalamus. No study has investigated the effect of GEB on alleviating type 2 diabetic symptoms. It is difficult to speculate whether GEB may be a hypoglycemic agent in non-obese type 2 diabetic animals, which represent the characteristics of type 2 diabetes in Asians.

We hypothesized that long-term administration of GEB would have a hypoglycemic effect by improving insulin resistance and β-cell function in a non-obese type 2 diabetic animal model. We tested the hypothesis by determining peripheral insulin resistance using the euglycemic hyperinsulinemic clamp assay and measuring insulin secretion capacity using the hyperglycemic clamp assay in partially pancreatectomized (Px) rats, a non-obese and insulin-insufficient type 2 diabetic animal model. We also measured insulin signaling in the hypothalamus to determine brain insulin sensitivity. Px rats are well represented the clinical characteristics of Asian type 2 diabetes.

## 2. Materials and Methods

### 2.1. Water Extract of GEB and Its Gastrodin and P-Hydroxybenzyl Alcohol Contents

GEB (100 g) was obtained from the Muju *Gastrodia elata* Blume Cooperative Production Co. (Muju, Korea), washed, dried at room temperature, freeze-dried, and powdered. The powder was extracted in distilled water at 90 °C for 12 h or in 70% ethanol at 70 °C for 12 h and both the extracts were centrifuged at 10,000× *g* at 4 °C for 20 min. The supernatants were lyophilized in a freeze-dryer. 

We measured total phenolic compound contents in the water extracts using Folin–Ciocalteu reagent and expressed them as mg gallic acid equivalents·g^−1^ [10]. Total flavonoid contents were measured using a method modified from the modified Davis method, and rutin was used as the standard [10]. The extracts were dissolved in methanol, and a syringe filter was used to remove undissolved contents. Gastrodin and *p*-hydroxybenzyl alcohol in the extract were analyzed by high-performance liquid chromatography using a Luna C18 column (4.6 mm × 250 mm; ID, 5 µm). The mobile phase solvents were acetonitrile and 0.1% formic acid in water (6:4, v:v) with isocratic elution at a flow rate of 1 mL/min, 40 °C in-column temperature, and UV detection at 270 nm. We used gastrodin and *p*-hydroxybenzyl alcohol standards to quantify the unknowns.

### 2.2. Animals and Ethics

Eight-week-old male Sprague–Dawley rats (weight, 218 g ± 23 g) were housed individually in stainless steel cages in a controlled environment (23 °C; 12-h light/dark cycle). All surgical and experimental procedures were performed according to the guidelines of the Animal Care and Use Review Committee of Hoseo University, Korea. The rats underwent a 90% pancreatectomy using the Hosokawa technique [11] under anesthesia induced by intramuscular injection of a mixture of ketamine and xylazine (100 mg/kg and 10 mg/kg body weight, respectively). The Px rats exhibited characteristics of type 2 diabetes (random glucose levels >180 mg/dL) [11,12].

### 2.3 Experimental Design

The GEB was provided to the Px rats in diets containing 0.5% or 2% of the GEB water extract. A total of 64 Px rats were assigned randomly to the following four groups, which differed according to diet: (1) 0.5% GEB; (2) 2% GEB; (3) 2% dextrin (Px-control); and (4) rosiglitazone (20 mg/kg body weight). All experimental animals were given free access to water and a high-fat diet containing either the assigned extracts or dextrose for eight weeks. The high-fat diet was a modified semi-purified AIN-93 formulation for experimental animals [13] that consisted of 40% carbohydrate, 20% protein, and 45% fat. The major carbohydrate, protein, and fat sources were starch and sugar, casein (milk protein), and lard (CJ Co., Seoul, Korea), respectively.

Overnight fasted serum glucose levels, food and water intake, and body weights were measured every week. An oral glucose tolerance test (OGTT) was performed at the seventh week in overnight-fasted animals by orally administering 2 g glucose/kg body weight [9]. Serum glucose and insulin levels were analyzed with a Glucose Analyzer II (Beckman-Coulter, Palo Alto, CA, USA) and radioimmunoassay kits (Linco Research, Billerica, MA, USA), respectively.

### 2.4. Energy Expenditure by Indirect Calorimetry

Energy expenditure was assessed at the beginning of the dark phase of the light–dark cycle after 6 h fasting at the seventh week of the experimental period. The rats were put into the metabolic chambers (airflow = 800 mL/min) with a computer-controlled O_2_ and CO_2_ measurement system (Biopac Systems Inc., Goleta, CA, USA) to measure their calorimetric parameters. The respiratory quotient (RQ) and resting energy expenditure (REE) were calculated using the equations described by Niwa *et al.* [14]. Oxygen consumption (VO_2_) and carbon dioxide production (VCO_2_) were measured over periods of 30 min. Data were averaged over 1 min intervals and VO_2_ and VCO_2_ values were adjusted for metabolic body size (kg^0.75^) [15]. Carbohydrate and fat oxidation were calculated from non-protein oxygen consumption as were their relative oxidative proportions and the amount of oxygen consumed per gram of substrate oxidized using RQ [15].

### 2.5. Euglycemic Hyperinsulinemic Clamp

A euglycemic hyperinsulinemic clamp was performed on 10 fasted, conscious rats after catheterization during week seven to determine insulin resistance, as described previously [16,17]. [3-^3^H] glucose (NEN Life Sciences, Boston, MA, USA) was infused continuously over 4 h at a rate of 0.05 μCi/min. Basal hepatic glucose output was measured in blood collected 100 and 120 min after initiating the [3-^3^H] glucose infusion. Then, a primed continuous infusion of regular human insulin was initiated at a rate of 20 pmol·kg^–1^·min^–1^ to raise plasma insulin concentration to approximately 1100 pM at 210–240 min. Serum glucose levels were made to approximately 6 mM by infusing glucose solution at various rates into the jugular vein. Rates of whole-body glucose uptake and basal glucose turnover were determined as the ratio of the [^3^H] glucose infusion rate to specific activity of plasma glucose (dpm/µmol) during the final 30 min of the respective experiments after measuring wet and dry [3-^3^H]glucose concentrations in plasma and tissues [16,17]. Hepatic glucose production in the hyperinsulinemic clamped state was determined by subtracting glucose infusion rate from whole-body glucose uptake. In addition, glucose uptake in the liver and skeletal muscles was measured by [^3^H] uptake [16,17].

### 2.6. Hyperglycemic Clamp

After seven weeks of treatment, all rats had catheters surgically implanted into the right carotid artery and left jugular vein under anesthesia with ketamine and xylazine. A hyperglycemic clamp was performed in 10 free-moving and overnight-fasted rats/group after 5–6 days of implantation to determine insulin secretion capacity, as described previously [11,18,19]. During the clamp, glucose was infused to maintain a serum glucose level of 5.5 mM above baseline, and serum insulin concentrations were measured at designated times. After the clamp, the rats were freely provided food and water for two days and then deprived of food for 16 h the next day. The rats were anesthetized with a mixture of ketamine and xylazine, and regular human insulin (5 U/kg body weight; Humulin; Eli Lilly, Indianapolis, IN, USA) was injected through the inferior vena cava. The rats were euthanized by decapitation 10 min later, and tissues were collected rapidly, frozen in liquid nitrogen, and stored at −70 °C for further experiments.

### 2.7. Immunoblot Analysis

Hypothalami collected from six rats stimulated with insulin for 10 min were lysed with RIPA lysis buffer containing protease inhibitors. After measuring protein contents in the lysate using the Bio-Rad protein assay kit (Hercules, CA, USA), lysates with equivalent amounts of protein (30–50 μg) were resolved by sodium dodecyl sulfate-polyacrylamide gel electrophoresis and immunoblotted with antibodies to phosphorylated Akt^ser478^, Akt, phosphorylated glycogen synthase kinase (GSK)-1β, and GSK-1β (Cell Signaling Technology, Beverly, MA, USA), [16,18]. The intensity of protein expression was determined using Imagequant TL (Amersham Biosciences, Piscataway, NJ, USA). These experiments were repeated three times for each group.

### 2.8. Immunohistochemistry

Five rats from each group were injected with BrdU (100 µg/kg body weight) after 6 weeks of treatment. The rats were anesthetized intraperitoneally 6 h post-injection with a mixture of ketamine and xylazine, and the brain and pancreas were dissected immediately, perfused with saline and a 4% paraformaldehyde solution (pH 7.2), sequentially, and post-fixed with the same fixative overnight at room temperature [20].

Two serial 5-μm paraffin-embedded tissue sections were selected from the seventh or eighth sections to avoid counting the same islets twice when measuring the β-cell area, BrdU incorporation, and apoptosis, as described previously using an immunohistochemistry method [20]. Endocrine β-cells were identified by applying guinea pig anti-insulin and rabbit anti-glucagon antibodies to the sections. Pancreatic β-cell area was measured by examining all non-overlapping images in two insulin-stained sections from each rat at 10× magnification with a Zeiss Axiovert microscope (Carl Zeiss Microimaging, Thornwood, NY, USA). Pancreatic β-cell mass, individual β-cell size, β-cell proliferation by BrdU incorporation, and apoptotic β-cell were measured as described previously [20].

### 2.9. Insulin Secretion in Mouse Insulinoma Cells (Min6 Cells)

Min6 cells were grown as previously described by Park *et al.* [21] in a 24 well plate at 6 × 10^4^ cells per well with high glucose DMEM containing 0.3% bovine serum albumin (BSA) and either vehicle (DMSO) or 20 μM of gastrodin, 4-hydroxybenzyl alcohol, and 4-hydroxybenzaldehyde for 16 h. Exendin-4 (2.5 nM; Sigma Co., St. Louis, MO, USA) was used as a positive control. After washing the cells with PBS, the Min6 cells were treated with 20 μM respective bioactive compounds in low (2 mM) or high glucose (20 mM) Krebs–Ringer–Hepes buffer containing 20 mM Hepes pH 7.4 and 5 mg∙mL^−1^ BSA for 30 min. Insulin concentrations in supernatants from all wells were measured using a radioimmunoassay kit (Linco Research, St. Charles, MO, USA).

### 2.10. Statistical Analyses

All data are expressed as means ± standard deviations, and all statistical analyses were performed using SAS ver. 9.1 (SAS Institute, Cary, NC, USA). Significant differences among the groups in both the animal study and cell-based study were identified with one-way analyses of variance. Significant differences in the main effects among the groups were detected using *post-hoc* Tukey’s tests. A *p*-value < 0.05 was considered significant.

## 3. Results

### 3.1. Bioactive Compounds in GEB

Total polyphenol and total flavonoid contents were slightly higher in the 70% ethanol GEB extracts than in the water extracts (Table 1). However, gastrodin and *p*-hydroxybenzyl alcohol contents, which are major bioactive compounds in GEB, were slightly higher in the water extracts (Table 1). Thus, we used the water extracts for the animal study.

### 3.2. Hypothalamic Insulin Signaling

**Figure 1:**
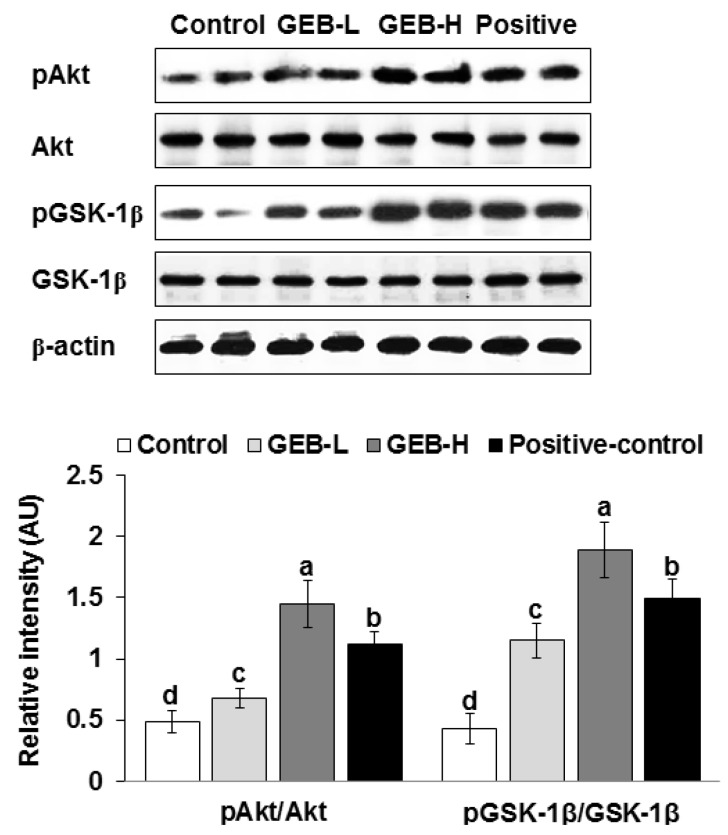
Hypothalamic insulin signaling at the end of experiment. Px rats were fed a high-fat diet supplemented with (1) 0.5% water extract of *Gastrodia elata* Blume (GEB-L); (2) 2% water extract of *Gastrodia elata* Blume (GEB-H); (3) 2% dextrin (Px-control); or (4) rosiglitazone (20 mg/kg body weight; positive-control) for eight weeks. Bars and error bars represented means ± standard deviation (*n* = 6). ^a,b,c,d^ Means of the bars without a common alphabet differed significantly at *p* < 0.05 by Tukey test.

GEB improved hypothalamic insulin signaling in a dose-dependent manner. GEB increased Akt and GSK-1β phosphorylations compared to those of the control (Figure 1). Therefore, the stimulation of hypothalamic insulin signaling promoted glucose homeostasis in the peripheries.

### 3.3. Energy Intake or Expenditure

Body weight and epididymal fat contents did not differ among groups (Table 2). GEB and rosiglitazone administration did not affect daily energy intake or expenditure compared to those of the control, although they were slightly lower in the control (Table 2). However, GEB increased carbohydrate oxidation and decreased fat oxidation compared to those of the control. The positive control showed similar patterns to those of the GEB group but was less efficacious than GEB (Table 2).

### 3.4. Glucose Tolerance

**Figure 2:**
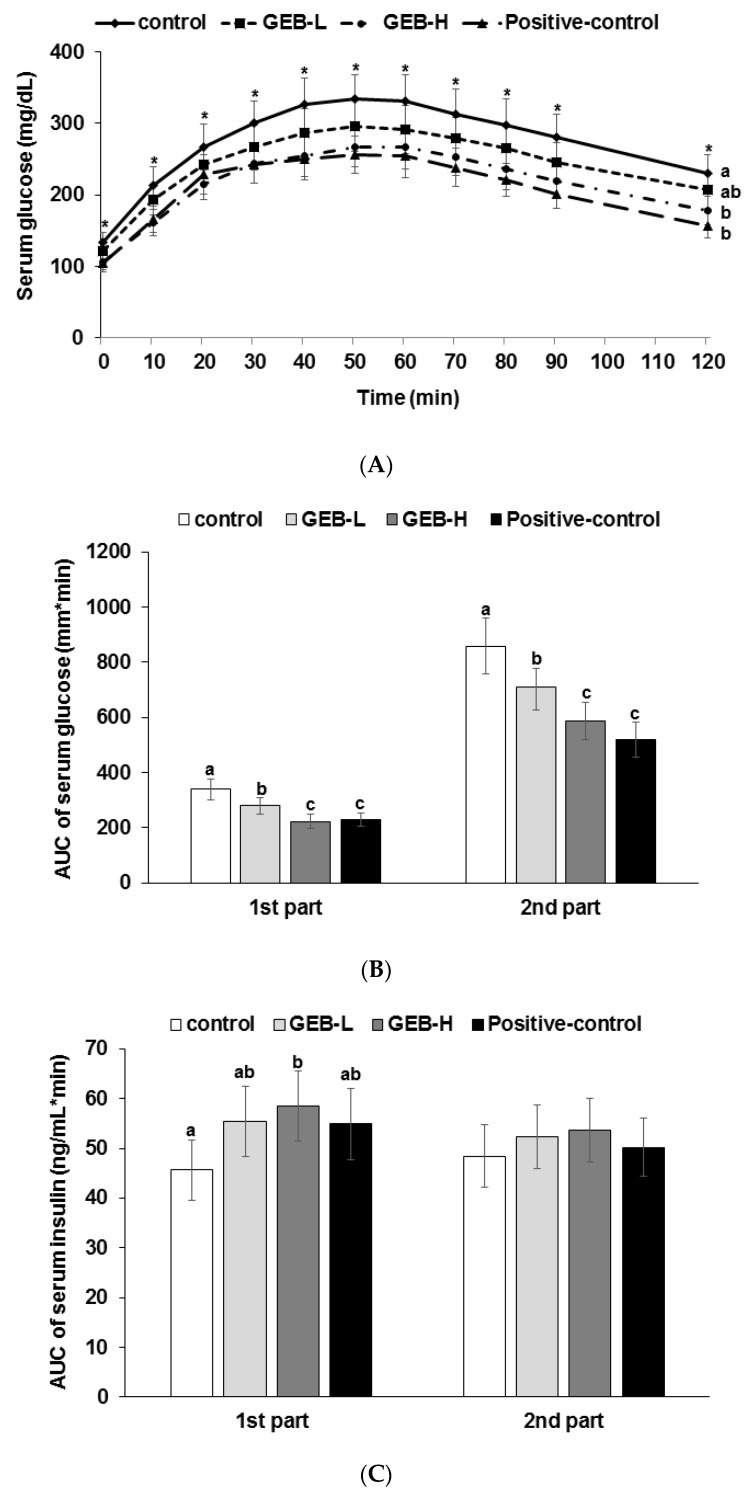
The changes in serum glucose levels (**A**) and the area under the curve for serum glucose (**B**) and insulin (**C**) during oral glucose tolerance testing. Px rats were fed a high-fat diet supplemented with (1) 0.5% water extract of *Gastrodia elata* Blume (GEB-L); (2) 2% water extract of *Gastrodia elata* Blume (GEB-H); (3) 2% dextrin (Px-control); or (4) rosiglitazone (20 mg/kg body weight; positive control) for eight weeks following oral loading with 2 g glucose per kg body weight. Bars and error bars represent means ± standard deviation (*n* = 16). ^a,b,c^ Means of the bars without a common alphabet differ significantly at *p* < 0.05 by Tukey test.

GEB lowered serum glucose levels dose-dependently at the peak 40–50 min after oral administration of 2 g glucose/kg body weight compared to the control (Figure 2A). Peak serum glucose concentrations decreased in all groups, except serum glucose concentrations at 120 min were lower in descending order of the control, GEB-L, GEB-H, and positive control groups (Figure 2B). GEB lowered the area under the curve (AUC) of serum glucose at the first and second parts during OGTT to a level lower than the control in a dose-dependent manner, and GEB-H exhibited similar efficacy to that of rosiglitazone (Figure 2B). The decrease in serum glucose concentrations during the OGTT was associated with serum insulin concentrations and insulin sensitivity. GEB-H increased AUC of serum insulin concentrations at the first part of the OGTT compared to the control but not in the second part (Figure 2C). Thus, GEB-H decreased serum glucose concentrations during OGTT by increasing serum insulin concentrations in the first part and by improving insulin sensitivity in the second part. GEB-H prevented the exacerbation of glucose tolerance, similar to rosiglitazone.

### 3.5. Insulin Sensitivity at a Hyperinsulinemic State

**Figure 3:**
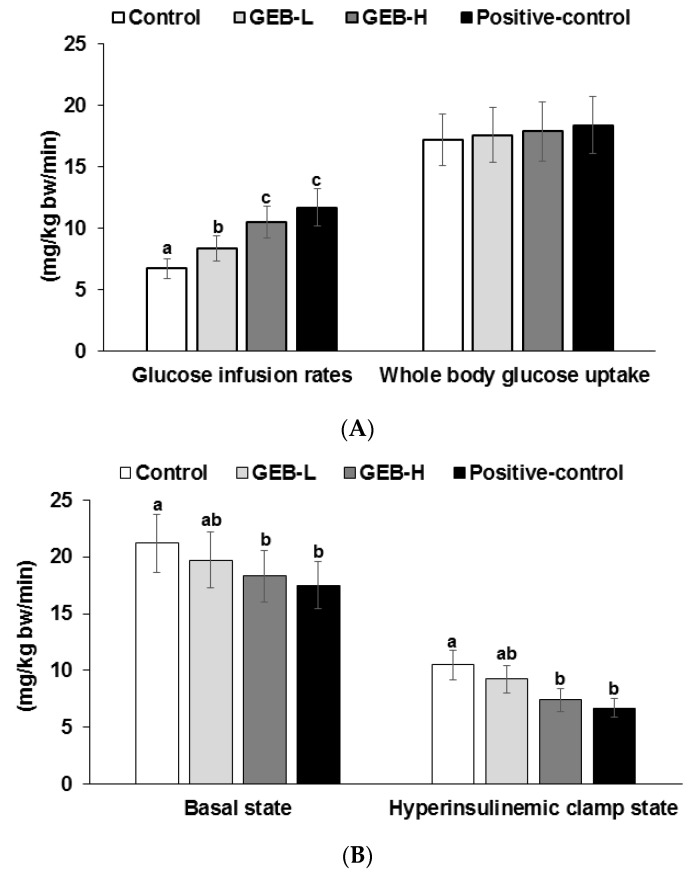

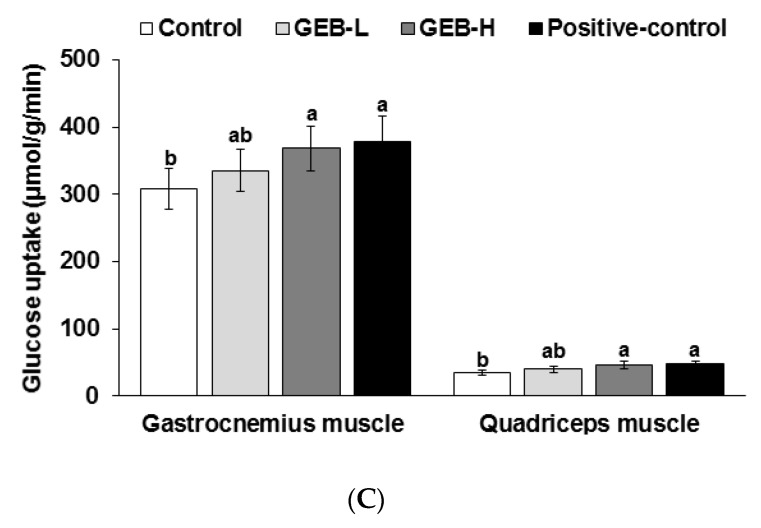
Metabolic parameters during euglycemic hyperinsulinemic clamp. Px rats were fed a high-fat diet supplemented with (1) 0.5% water extract of *Gastrodia elata* Blume (GEB-L); (2) 2% water extract of *Gastrodia elata* Blume (GEB-H); (3) 2% dextrin (Px-control); or (4) rosiglitazone (20 mg/kg body weight; positive control) for eight weeks. Whole body glucose infusion rates (GIR), glucose uptake (**A**); and hepatic glucose output at basal and clamped states (**B**) were determined; The glucose uptake in the gastrocnemius and quadriceps muscles was also measured in a hyperinsulinemic state (**C**). Bars and error bars represent means ± standard deviation (*n* = 8). ^a,b,c^ Means of the bars without a common alphabet differ significantly at *p* < 0.05 by Tukey test.

Insulin resistance was determined during the hyperinsulinemic euglycemic clamp. Glucose infusion rates were higher in rosiglitazone and GEB-H groups than the control group (Figure 3A). However, whole-body glucose uptake did not differ among groups. Hepatic glucose output during the hyperinsulinemic clamp state was lower in the GEB-H than in the control group, but the decrease was not as great as that observed in the rosiglitazone group (Figure 3B). Hepatic glucose output in the basal state was lower in the GEB-H and rosiglitazone group than in the control (Figure 3B). These results suggest that GEB-H improved whole-body insulin resistance and hepatic insulin resistance in the basal and hyperinsulinemic clamp states, but that the improvement was less than that observed in response to rosiglitazone.

The glucose uptake in gastrocnemius and quadricep muscles during hyperinsulinemic euglycemic clamp was lower in the control group and it was higher in the GEB-H group. The increase was similar to the positive control group (Figure 3C).

### 3.6. Glucose-Stimulated Insulin Secretion

**Figure 4:**
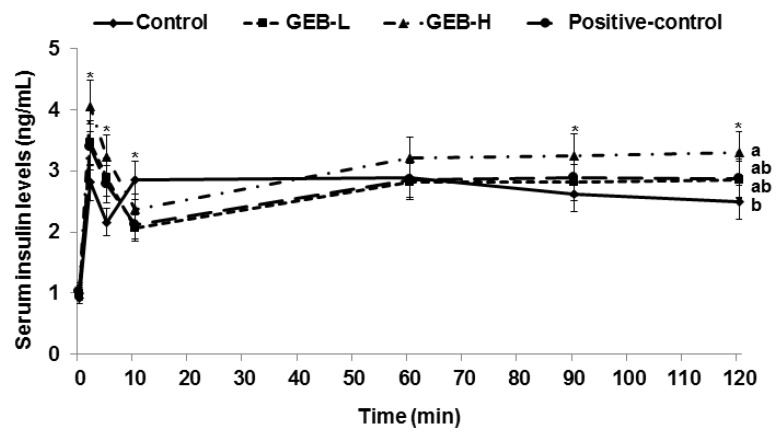
Insulin secretion capacity during hyperglycemic clamp. Px rats were fed a high fat diet supplemented with (1) 0.5% water extract of *Gastrodia elata* Blume (GEB-L); (2) 2% water extract of *Gastrodia elata* Blume (GEB-H); (3) 2% dextrin (Px-control); or (4) rosiglitazone (20 mg/kg body weight; positive control) for eight weeks. During hyperglycemic clamp, serum insulin levels were measured as serum glucose levels at 5.5 mM above fasting levels were maintained. Dots and error bars represent means ± standard deviation (*n* = 8). * Significantly different among the different four groups at *p* < 0.05 by one-way ANOVA.

Glucose-stimulated insulin secretion was determined by measuring serum insulin level when serum glucose level increased by 100 mg/dL from baseline, which is called the hyperglycemic clamp. Serum insulin levels peaked between 2 and 5 min during the hyperglycemic clamp, and then declined to a nadir at 10 min, which was the first phase of insulin secretion (Figure 4). We observed an ascending second phase of serum insulin at 60–120 min in all rats. The first phase of insulin secretion was lower in descending order of GEB-H, GEB-L, rosiglitazone, and control (Table 3). However, the second phase of insulin secretion was greater only in the GEB-H group compared to that in the other groups (Table 4). Thus, GEB, but not rosiglitazone, was a good insulin secretagogue to potentiate glucose-stimulated insulin secretion.

Glucose infusion rates during the hyperglycemic clamp are determined as the balance of β-cell function and insulin sensitivity during a hyperglycemic state [18]. Insulin sensitivity at the hyperglycemic state is calculated as the ratio of glucose infusion rates to steady-state serum insulin concentration [18]. The glucose infusion rate needed to maintain serum glucose level 5.5 mM above the baseline increased in ascending order of the control, GEB-L, GEB-H, and rosiglitazone groups (Table 3). Insulin sensitivity during the hyperglycemic state was highest in the rosiglitazone group but also increased in a dose-dependent manner in the GEB group compared to that in the control (Table 3). Therefore, GEB-H improved insulin sensitivity in a hyperglycemic state, but not as much as rosiglitazone.

### 3.7. Pancreatic β-Cell Mass, Proliferation, and Apoptosis

Pancreatic β-cell area is associated with the number and individual size of β-cells. Larger individual β-cell size indicates hypertrophy due to increased insulin resistance. Thus, pancreatic β-cell area should be elevated by the number of β-cells to improve diabetic status. Although pancreatic β-cell area did not differ between the control and positive-control groups, individual β-cell size was smaller in the positive-control and GEB-H groups than in the control group (Table 4).

GEB increased pancreatic β-cell area in a dose-dependent manner, which was associated with reduced individual β-cell size and increased β-cell number (Table 4). Pancreatic β-cell mass was calculated by multiplying β-cell area by pancreatic weight. Pancreatic β-cell mass was lower in the control group than in the positive-control group, whereas GEB increased pancreatic β-cell mass, dose-dependently (Table 4).

β-cell number is related to the net of β-cell proliferation and β-cell apoptosis. The Px control rats had a higher frequency of β-cell apoptosis than that in the positive control rats, but we detected no significant difference in β-cell proliferation between the control and positive control groups (Table 4). GEB-H increased the number of β-cells (hyperplasia) by enhancing proliferation and reducing apoptosis in the Px rats (Table 4). Therefore, GEB increased β-cell mass by elevating β-cell proliferation and decreasing β-cell apoptosis.

### 3.8. Glucose-Stimulated Insulin Secretion with Gastrodin, 4-Hydroxybenzyl Alcohol, and 4-Hydroxybenzaldehyde

**Figure 5:**
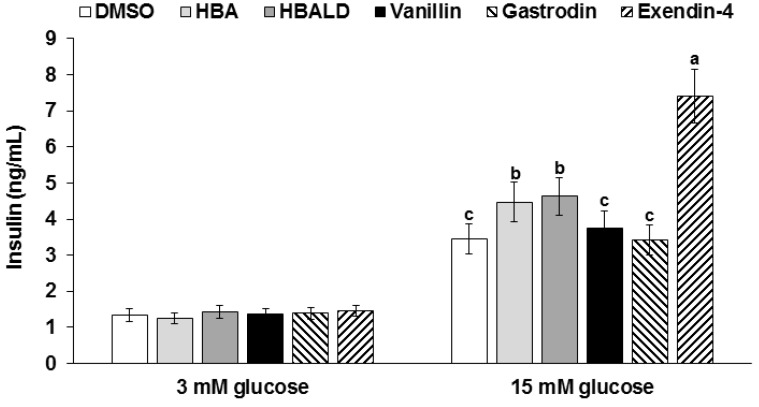
Glucose-stimulated insulin secretion in insulinoma Min6 cells. Min6 cells were treated with low (2 mM) and high glucose (20 mM) Krebs-Ringer-Hepes buffer with 20 μM gastrodin, 4-hydroxybenzyl alcohol, and 4-hydroxybenzaldehyde for 30 min. The positive control was 2.5 nM exendin-4. Insulin levels in the supernatant were measured by RIA kit. Values are means ± SD (*n* = 5). ^a,b,c^ Means of the bars without a common alphabet significantly differ at *p* < 0.05 by Tukey test.

Gastrodin, 4-hydroxybenzyl alcohol, and 4-hydroxybenzaldehyde do not stimulate insulin secretion with a 2 mM glucose solution (low glucose) in insulinoma cells, but 20 µM 4-hydroxybenzyl alcohol and 20 µM 4-hydroxybenzaldehyde potentiated insulin secretion by 1.2 and 1.3-fold compared to that by the DMSO treatment, respectively, in a 20 mM glucose media (high glucose) (Figure 5). However, the increase was not as much as exendin-4. Thus, GEB might improve glucose-stimulated insulin secretion, possibly by 4-hydroxybenzyl alcohol and 4-hydroxybenzaldehyde.

## 4. Discussion

The brain, particularly the hypothalamus, plays an important role regulating energy and glucose metabolism [22]. In addition, Alzheimer’s disease and type 2 diabetes share impaired insulin signaling [7]. Alzheimer’s disease is associated with hippocampal insulin resistance, whereas type 2 diabetes develops due to insulin resistance in peripheral tissues, including the liver, adipose tissue, and islets [7,23]. Thus, insulin signaling in the hypothalamus and hippocampus are connected to peripheral glucose metabolism. GEB may improve type 2 diabetic symptoms as it has been traditionally used for brain-related diseases, such as convulsions, ischemia, Alzheimer’s disease, and tremors. The purpose of the study was to investigate whether long-term consumption of GEB would improve type 2 diabetic symptoms by reducing insulin resistance and potentiating β-cell function and mass in partial Px rats.

When Asians increase insulin resistance, they exhibit normal insulin levels or hypoinsulinemia and easily progress from glucose intolerance to type 2 diabetes [24]. Px rats are a good model to examine the relationship between β-cell function and insulin resistance and β-cell expansion since they are a non-obese and insulin-insufficient type 2 diabetic model with characteristics relevant to Asian type 2 diabetes. Px rats exhibit a similar phenotype to Asian type 2 diabetes [24,25]. After removing 90% of the pancreas, the pancreas regenerates up to 40%–50% of the intact pancreas and insulin secretion capacity is about 50%–60% of the non-diabetic rats [18,26]. Overnight-fasting serum glucose levels were over 130–150 mg/dL and post-prandial serum glucose levels about 230–280 mg/dL. They showed moderate diabetic status. Thus, Px rats are non-obese and insulin insufficient type 2 diabetic animal model.

GEB has been reported to have neuroprotective activities to prevent and/or alleviate dizziness, epilepsy, stroke, and dementia. GEB contains small compounds, such as gastrodin, 4-hydroxybenzyl alcohol, 4-hydroxybenzaldehyde, 4-hydroxy-3-methoxybenzaldehyde, and vanillin, which can pass through the BBB, suggesting that it acts in the brain [6]. The brain, particularly the hypothalamus, regulates energy and glucose metabolism [7]. The brain receives neural inputs of glucose status from the periphery, and neurons directly sense glucose levels. When glucose levels rise, glucose-responsive neurons increase firing through an ATP-sensitive K^+^ channel, whereas glucose-sensitive neurons decrease firing [22]. Both neurons are related to changes in food intake, sympathoadrenal activity, and energy expenditure in the states of extreme hyperglycemia and hypoglycemia [27]. Glucose responsive neurons are hyper-responsive to glucose in rats with diet-induced obesity and insulin-dependent diabetes [27]. Thus, brain glucose sensing differs in obese and diabetic states. Modulation of brain glucose metabolism can relieve the symptoms of obesity and type 2 diabetes [28]. As GEB has actions against dementia, which is associated with brain insulin resistance, GEB may alter brain insulin resistance. Brain insulin resistance is also associated with peripheral insulin resistance, particularly in the liver [23]. Therefore, GEB may influence peripheral glucose metabolism in patients with type 2 diabetes. In the present study, GEB potentiated hypothalamic insulin signaling (pAkt → pGSK-1β) in parallel with improving hepatic insulin resistance in partial Px rats. GEB may regulate peripheral glucose metabolism through brain insulin signaling.

GEB is known to improve energy metabolism possibly by enhancing leptin signaling [29]. In our previous study GEB reduced weight gain and epididymal fat pads in diet-induced obese rats by improving hypothalamic leptin signaling and reducing NPY and AgRP expression [9]. However, the present study GEB did not lower in body weight and epididymal fat pads in diabetic Px rats, although GEB tended to reduce daily energy intake and increase daily energy expenditure. This discrepancy between two studies was related to differences in the animal models. Diabetic Px rats showed hyperglycemia with insulin insufficiency in the present study, whereas diet-induced obese rats exhibited hyperinsulinemia with normoglycemia. Diabetic Px rats are non-obese rats and they lose weight gain when the diabetic symptoms are severe. Although GEB improved energy metabolism in both studies, it maintained body weight and body fat in diabetic Px rats by enhancing glucose utilization, possibly due to decreased urinary glucose excretion.

Type 2 diabetes is induced by impaired insulin sensitivity and secretion. When insulin resistance is initially induced in the liver, skeletal muscles, and adipose tissues by certain conditions, such as obesity, stress, inflammation, and aging; however, insulin secretion from pancreatic β-cells is sufficient to maintain normoglycemia and, thus, compensates for insulin resistance [1]. Insulin insufficiency leads to type 2 diabetes in an increased insulin-resistant state. The present study showed that whole-body glucose infusion rates representing whole-body insulin resistance increased. Increased glucose infusion rates in GEB-H without whole body glucose uptake was related to decreased hepatic glucose output, indicating that GEB-H was lower hepatic insulin resistance. Surprisingly, glucose uptake in skeletal muscles was higher in GEB-H than the control although whole body glucose uptake slightly increased but not significantly different. The proportion of glucose uptake in skeletal muscles may not be large enough to influence whole body glucose uptake. These results suggested that GEB influenced glucose metabolism in both the liver and skeletal muscles but GEB improved insulin sensitivity in the liver more than skeletal muscles. 

In this study, Px rats had 50%–60% glucose-stimulated insulin secretion capacity of the rats with an intact pancreas [18,26]. However, as the decrease in insulin secretion itself takes longer to induce type 2 diabetes, a high-fat diet was fed to exacerbate glucose homeostasis and require more insulin secretion by increasing insulin resistance [20]. Thus, the potentiation of glucose-stimulated insulin secretion is necessary to improve glucose homeostasis in partial Px rats. As Asians have lower insulin secretion capacity [2], partial Px rats are an optimal model with which to explore therapeutic herbs for treating Asians. Our results show that GEB potentiated glucose-stimulated insulin secretion. GEB has been shown to enhance phosphorylation of both phosphatidylinositol 3-kinase (PI3K) and cAMP-responsive element binding protein (CREB) and to increase brain-derived neurotrophic factor in HT22 hippocampal cells. As islets are also neuronal cells, GEB may increase PI3K and CREB phosphorylation [30], which is involved in glucose-stimulated insulin secretion. In addition, administering 4-hydroxybenzyl methyl ether (10 mg/kg, p.o.) isolated from GEB, significantly increases phosphorylation of cortical and hippocampal protein kinase A (PKA)/CREB [31]. The present study showed that GEB might improve glucose-stimulated insulin secretion, possibly by 4-hydroxybenzyl alcohol and 4-hydroxybenzaldehyde, and the pathway to potentiate insulin secretion might be similar to that used by neurons.

Potentiated insulin secretion should be combined with increased β-cell mass because β-cell mass plays an important role in maintaining insulin secretion. Sulphonylureas have been used to treat diabetes by increasing insulin secretion by closing K_ATP_-channels to trigger calcium influx [32]. They release insulin not only under high glucose but also under low glucose conditions without increasing β-cell mass [32]. Eventually, sulphonylureas fail to normalize serum glucose levels. However, new insulin secretagogues, such as exenatide, not only induce significant increases in serum insulin but also increase the number of insulin- and GLP-1-positive cells [33]. GLP-1 receptor agonists, such as exenatide, lower glucose concentration by augmenting insulin secretion and suppressing glucagon release [34]. In addition, exenatide maintains β-cell mass by protecting β-cell apoptosis [34]. Exenatide activates the cAMP, PKB, and PKA signal transduction pathways to activate PI3K and its downstream mitogen-activated protein kinase/extracellular regulated kinase. Thus, GLP-1 receptor agonists are better insulin secretagogues than sulphonylureas. Our results show that GEB increased glucose-stimulated insulin secretion by increasing β-cell mass and proliferation. Thus, GEB might be a good hypoglycemic agent, particularly for treating non-obese type 2 diabetes with insulin insufficiency.

## 5. Conclusions

GEB enhanced hypothalamic insulin signaling in a dose-dependent manner and it was associated with improving whole body and hepatic insulin sensitivity. In addition, GEB potentiated glucose-stimulated insulin secretion by increasing β-cell mass and enhanced peripheral insulin resistance in a non-obese and insulin-insufficient type 2 diabetic animal model. Therefore, GEB is a therapeutic candidate for preventing and alleviating type 2 diabetes in Asian patients.

## Figures and Tables

**Table 1:** The contents of gastrodin and *p*-hydroxybenzyl alcohol in GEB.

	Water Extracts (*n* = 3)	70% Ethanol Extracts (*n* = 3)
Total phenols	755 ± 17.3	784 ± 19.1 *
Total flavonoids	68.3 ± 2.3	69.2 ± 3.5
Gastrodin	3.78 ± 0.016	3.69 ± 0.01
*p*-hydroxybenzyl alcohol	0.45 ± 0.0002	0.43 ± 0.0001

* Significantly different from water extract at *p* < 0.05.

**Table 2:** Energy metabolism at the end of experimental periods.

	Control (*n* = 16)	GEB-L (*n* = 16)	GEB-H (*n* = 16)	Positive-Control (*n* = 16)
Body weight (g)	324 ± 28	336 ± 29	345 ± 30	333 ± 29
Epididymal fat pads (g)	4.9 ± 0.9	5.0 ± 0.8	5.2 ± 0.9	5.2 ± 0.7
Caloric intake (kcal/day)	86.6 ± 9.5	83.4 ± 9.6	80.5 ± 9.2	79.6 ± 8.8
Caloric expenditure (kcal/kg bw^0.75^/day)	104 ± 11	108 ± 12	111 ± 11	111 ± 12
Carbohydrate oxidation (kcal/kg bw^0.75^/day)	4.4 ± 0.6 ^c^	5.5 ± 0.6 ^a,b^	6.1 ± 0.7 ^a^	5.3 ± 0.6 ^b^
Fat oxidation (kcal/kg bw^0.75^/day)	6.7 ± 0.7 ^a^	5.9 ± 0.7 ^a,b^	5.7 ± 0.7 ^b^	6.5 ± 0.7 ^a^

Values are means ± standard deviation. Px rats were fed a high fat diet supplemented with (1) 0.5% water extract of *Gastrodia elata* Blume (GEB-L); (2) 2% water extract of *Gastrodia elata* Blume (GEB-H); (3) 2% dextrin (Px-control); or (4) rosiglitazone (20 mg/kg body weight; positive control) for eight weeks. ^a,b,c^ Means on the same row with different superscripts were significantly different at *p* < 0.05.

**Table 3:** Insulin secretion capacity during hyperglycemic clamp.

	Control (*n* = 8)	GEB-L (*n* = 8)	GEB-H (*n* = 8)	Positive-Control (*n* = 8)
Overnight fasted serum glucose (mg/dL)	132 ± 16 ^a^	120 ± 17 ^a,b^	108 ± 14 ^b^	106 ± 11 ^c^
Overnight fasted serum insulin (ng/mL)	0.97 ± 0.11	0.99 ± 0.14	1.05 ± 0.12	1.04 ± 0.11
Area under the curve of serum insulin at first phase (ng/mL·min)	20.7 ± 2.1 ^c^	24.7 ± 3.0 ^b^	29.4 ± 3.1 ^a^	20.5 ± 2.7 ^c^
Area under the curve of serum insulin at second phase (ng/mL·min)	258 ± 28 ^b^	254 ± 27 ^b^	291 ± 30 ^a^	240 ± 27 ^b^
Glucose infusion rate (mg/kg bw/min)	8.3 ± 1.0 ^c^	10.6 ± 1.2 ^b^	13.4 ± 1.5 ^a^	12.7 ± 1.5 ^a^
Insulin sensitivity (µmol glucose·min^−1^·100 g^−1^ per µmol insulin/L)	12.5 ± 1.4 ^d^	14.6 ± 1.6 ^c^	16.4 ± 1.7 ^b^	18.3 ± 1.7 ^a^

Values are means ± standard deviation. Px rats were fed a high-fat diet supplemented with (1) 0.5% water extract of *Gastrodia elata* Blume (GEB-L), (2) 2% water extract of *Gastrodia elata* Blume (GEB-H), (3) 2% dextrin (Px-control), or (4) rosiglitazone (20 mg/kg body weight; positive control) for eight weeks. ^a,b,c^ Means on the same row with different superscripts were significantly different at *p* < 0.05.

**Table 4:** The modulation of islet morphometry.

	Control (*n* = 8)	GEB-L (*n* = 8)	GEB-H (*n* = 8)	Positive-Control (*n* = 8)
β-cell area (%)	6.5 ± 0.8 ^b^	7.0 ± 0.9 ^a,b^	7.6 ± 0.9 ^a^	7.0 ± 0.9 ^a,b^
Individual β-cell size (μm^2^)	227 ± 28 ^a^	208 ± 26 ^a,b^	187 ± 25 ^b^	193 ± 27 ^b^
Absolute β-cell mass (mg)	18.7 ± 2.8 ^c^	23.3 ± 3.0 ^b^	28.8 ± 3.5 ^a^	23.8 ± 2.9 ^b^
BrdU^+^ cells (% BrdU^+^ cells of islets)	0.84 ± 0.10 ^b^	0.92 ± 0.12 ^b^	1.11 ± 0.12 ^a^	0.93 ± 0.12 ^b^
Apoptosis (% apoptotic bodies of islets)	0.70 ± 0.09 ^a^	0.65 ± 0.07 ^a,b^	0.59 ± 0.08 ^b^	0.61 ± 0.07 ^b^

Values are means ± standard deviation. Px rats fed a high fat diet supplemented with (1) 0.5% water extract of *Gastrodia elata* Blume (GEB-L); (2) 2% water extract of *Gastrodia elata* Blume (GEB-H); (3) 2% dextrin (Px-control); or (4) rosiglitazone (20 mg/kg body weight; positive-control) for 8 weeks. ^a,b,c^ Means on the same row with different superscripts were significantly different at *p* < 0.05.
